# The oligonucleotide frequency derived error gradient and its application to the binning of metagenome fragments

**DOI:** 10.1186/1471-2164-10-S3-S10

**Published:** 2009-12-03

**Authors:** Isaam Saeed, Saman K Halgamuge

**Affiliations:** 1 MERIT, Biomedical Engineering, Department of Mechanical Engineering, The University of Melbourne, Australia

## Abstract

**Background:**

The characterisation, or binning, of metagenome fragments is an important first step to further downstream analysis of microbial consortia. Here, we propose a one-dimensional signature, OFDEG, derived from the oligonucleotide frequency profile of a DNA sequence, and show that it is possible to obtain a meaningful phylogenetic signal for relatively short DNA sequences. The one-dimensional signal is essentially a compact representation of higher dimensional feature spaces of greater complexity and is intended to improve on the tetranucleotide frequency feature space preferred by current compositional binning methods.

**Results:**

We compare the fidelity of OFDEG against tetranucleotide frequency in both an unsupervised and semi-supervised setting on simulated metagenome benchmark data. Four tests were conducted using assembler output of Arachne and phrap, and for each, performance was evaluated on contigs which are greater than or equal to 8 kbp in length and contigs which are composed of at least 10 reads. Using both G-C content in conjunction with OFDEG gave an average accuracy of 96.75% (semi-supervised) and 95.19% (unsupervised), versus 94.25% (semi-supervised) and 82.35% (unsupervised) for tetranucleotide frequency.

**Conclusion:**

We have presented an observation of an alternative characteristic of DNA sequences. The proposed feature representation has proven to be more beneficial than the existing tetranucleotide frequency space to the metagenome binning problem. We do note, however, that our observation of OFDEG deserves further anlaysis and investigation. Unsupervised clustering revealed OFDEG related features performed better than standard tetranucleotide frequency in representing a relevant organism specific signal. Further improvement in binning accuracy is given by semi-supervised classification using OFDEG. The emphasis on a feature-driven, bottom-up approach to the problem of binning reveals promising avenues for future development of techniques to characterise short environmental sequences without bias toward cultivable organisms.

## Background

Metagenomics is a relatively recent field of research, dealing primarily with the investigation of microbial consortia of uncultivable organisms. It has enabled the study of microbiota sampled directly from environmental niches, such as the ocean [1,2], soil [3], hot springs [4], hydrothermal vents [5], polar ice caps [6] and hypersaline environments [7]. In depth investigation of these consortia have given insight into microbial ecology [8], diversity [9], as well as archeal lineages [10]. Not only is such knowledge valuable to the understanding of our biosphere, it has also facillatated advancement of biotechnology [11,12], human physiology [13] and sequencing of contaminated samples of now extinct species [14,15], to name a few. Prior to the metagenomics approach, we see that attempts at characterising microbial communities using pure clonal samples of constiuent organisms resulted in a low discovery rate of novel microbes [9].

Metagenomics is able to tackle this problem by means of direct sequencing of an environmental sample without the need for a pre-cloning step. This enables approximately 99% of Earth's undiscovered microbiota, which resist standard laboratory culturing techniques, to be sequenced and analysed. However, when an environmental sample is sequenced *en masse*, a fundamental computational challenge is introduced: the characterisation of sequenced reads with respect to their phylogenetic origin [16]. Such *in silico *profiling of sequenced DNA is referred to as binning.

Binning is an important first step to further downstream analysis of a metagenome. Of particular interest in this preliminary stage of analysis is the taxonomic composition of the sample, and further, the association between sequenced DNA fragments and their parent organism. Many reported attempts at this analysis are founded on one of three key concepts: marker gene based assignment, sequence similarity assignments or sequence composition based assignments.

### Taxonomic profiling using conserved marker genes

Through various stages of an organism's evolution changes take place in the composition its genome, permitting adaptation to changes in the environment, for example. Different locations in the genome experience distinct rates of change. Hyper-variant regions are particularly found in non-coding, inter-genic regions [17]. This is because pronounced changes in genes that code for particular functions will degrade characteristic functionality of an organism. The exceptions to this are slowly evolving marker genes in the guise of non-coding ribsomal RNAs. These conserved marker genes have been fundamental to the study of microbial phylogeny [18]. Prior to the discovery of such marker genes, phylogenetic analysis of microbes revealed the existence of only two primeval lineages. However, a founding study [19] highlighted the insuffciency of existing approaches to capture all extant lineages. It was this which lead to the establishment of three primary kingdoms or domains of Archea, Bacteria and Eucarya.

More recently, studies that adopt this approach have greatly contributed to our knowledge of the actual diversity of microorganisms [20-22]. Automatation of the phylotyping procedure for metagenomic DNA using 16S rRNA markers are also becoming more prevalent [23-27]. In [28] two fundamental questions are posed regarding the number of bacterial phylotypes that can co-exist and the way in which they are organised, and an attempt at addressing both has been through 16S rRNA sampling of a bacterioplankton assemblage. As [28] argues, these two pieces of information are critical to the understanding of function, population biology and biogeography. Further, [29] uses 16S rRNA libraries to compare the phylogenetic distance between various microbial communities.

On the one hand where such marker genes are providing valuable insight into the composition of microbial assemblages, they carry with them the inability to characterise the majority of sequenced reads. It has been reported that these marker genes appear infrequently in a typical set of sequenced reads, despite the high density of open reading frames found in microbial genomes [30]. Consequently, only a fraction of reads can be assigned.

Read length is also a factor to consider when attempting classification using marker genes. 16S rRNA gene are generally 1,500 base pairs in length and as such will be distributed over multiple reads of current state-of-the-art sequencing technologies. Complications then arise in taxonomic profiling when marker genes are assumed to be partially located on sequenced reads. However, this is not so much a critical issue for future sequencing platforms, as whole-molecule sequencing is designed to deliver sequenced reads that are in the order of length of the marker genes [31]. Marker genes will continue to be used for taxonomic characterisation of a metagenome, as it is arguably the most accurate [30].

### Characterisation based on similarity to known sequences

A viable alternative to the use of conserved regions of genomic DNA is the use of previously sequenced homologs as a basis for phylogenetic characterisation. Databases of complete genomes and information on genes, protein families and so forth have experienced exponential growth, especially over the last few decades, attributed in part to advances made by marker gene based profiling of microbial organisms. Tapping into this resource pool has been the focus of a variety of methods. For instance, early similarity methods used a simple BLAST (Basic Local Alignment Search Tool) search against databases of previously sequenced complete genomes to assign short fragments to a particular taxonomic rank, using homologs [32] or orthologs [33,34]. Currently, the length of reads is an important challenge [35] and methods that are able to assign short stretches of DNA are required, particularly where next generation sequencing platforms are used to sequence metagenomes. In fact, claims have even been made to a solution for phlyotyping a metagenome irrespective of the sequencing technique used [36].

The strength of these methods relies on the length of sequences able to be assigned. We see that recent attempts achieve accurate classification of reads down to 80 base pairs in length [37]. Despite this, for real metagenomic samples, fewer than 10% of the reads could be identified using the Pfam domain and protein families [38], which is suggestive of poor database coverage over extant microorganisms. Of this 10%, only a fraction could be assigned to a particular lineage.

In general, a bottleneck exists in conducting the initial search against various databases, often necessitating the use of a large number of CPU hours on high performance computing solutions. As these databases continue to grow at their current rate, this bottle-neck will increasingly impose significant delays in any metagenomic project, perhaps with minimal pay-off for the classification of novel organisms. However, by adopting these strategies we are fundamentally relying on the assumption that novel microbes sampled from the environment will be represented in existing databases. With an abundance of microbial diversity yet to be discovered, it is counter-intuitive to found decisions on previously discovered genomes and protein sequences [39], particularly when the majority of these are derived from culture-dependant techniques, while an estimated 99% of novel microbes are yet to be discovered can not be cultured using current *in vivo *techniques [36].

### Characterisation based on sequence composition

The composition of a DNA sequence is defined by the non-random ordering of its base-pairs, in terms of the four atomic nucleotides. Taking into account that there are specific causes and evolutionary factors for the variation in base composition of genomic DNA, methods have been developed to extract common patterns between organisms at varying levels of taxonomic resolution, such that sequences of similar species are able to be grouped. The general trend with compositional approaches has been the modification of machine learning methods to work around existing compositional feature spaces. Deterministic pattern spaces such as oligonucleotide frequency counts are among the more dominant of choices among compositional binning methods. These operate on the assumption that the relative abundance of certain words - also referred to as *oligos*, i.e. ACGTA is a 5-mer oligo - primarily dictate the association of one sequence to another. This is particularly useful for observing codon usage biases, for example. In fact, compositional biases, particularly in the case of tetranucleotide (4-mer) frequencies, is hypothesised to have strong biological significance in terms of phylogeny [40-42]. It is further argued that the larger number of permutations possible in tetranucleotide frequencies allows greater authority to discriminate between genomic fragments from different genomes. This argument holds if the conditions of low intragenomic variation and large sequence length (in this case a 40 kb [42] fosmid sized vector is used) hold. Due to the large number of combinations of oligos possible, tetranucleotide frequency has been reported to have greater discriminatory power than the G-C content of a sequence, for instance [42]. Clustering tetranucleotide frequencies using fixed-size Self-Organising Maps (SOMs) has been shown to be possible [43]. However, the imposition of a fixed size SOM has been attributed to feature maps that do not faithfully represent the input data, and as such the Growing Self-Organising Map has been used to alleviate this flaw [44-46].

In the context of current next generation sequencing technologies, we find that reads are as short as 80-100 base pairs. In light of this, methods that operate on nucleotide frequency alone are at a disadvantage, as a signal strong enough to make inferences on phylogenetic origin of a sequence requires long stretches of DNA. It has been shown that 40,000 bp is an acceptable sequence length to make accurate predictions [42], yet it has also been shown that sequences as short as 1,000 bp can be classified [16]. As yet, the 1,000 bp barrier - as it has been colloquially termed in the literature - is still an open challenge. To counteract this limitation, methods that adopt nucleotide frequencies as a means of sequence representation typically operate on assembled contigs. However, for complex communities the required amount of coverage for modest assembly translates to a substantial sequencing requirement. The feasibility, therefore, for current composition based methods looks to be limited to microbial consortia with minimal to moderate diversity. Further, these methods compensate for weak phylogenetic signals by consolidating information from other sources, such as external databases. Methods have also used training data from potential homologs from public databases to construct a representative signal for a particular clade [30]. In the same study it is also shown that it is possible to generate training data directly from the metagenome itself, but it is argued that at least 100 kbp of data is required to construct an accurate model for a particular organism at a particular taxonomic rank.

The literature suggests that significant advances in compositional approaches to the binning problem have primarily looked at the issue of representing the composition of a sequence, rather than refining machine learning methods that operate in a sub-par feature space. Such is the case with the succession of G-C content by tetranucleotide frequencies [42,47], for example. There is much that can be unveiled when patterns are extracted from DNA sequences, and since compositional methods are generally database-independent they are not susceptible to cloning biases observed in similarity-based methods. It is, however, a matter of knowing what patterns to extract and how best to extract them. Here we propose the use of the oligonucleotide frequency derived error gradient (OFDEG) as a feature space for the characterisation of DNA sequences from isloate organisms. The proposed feature space relies on the concept of oligonucleotide frequency profiles and their demonstrated ability to characterise genomic DNA.

## Results and discussion

### Evaluation on simulated metagenomic data sets

#### Data set description

Recently published metagenomic benchmark data sets [39] have been selected to evaluate the binning performance using our proposed DNA sequence representation. The benchmarks were formed using real DNA sequences of 113 isolate microbial genomes, sequenced at DOE Joint Genome Institute. The dominant strain in the simLC set is given a coverage of approximately 5.19× and just over 27 Mbp in total sequence length. The dominant strain is anked by 115 lower abundance strains, with coverage less than 1.2×. SimMC introduces three dominant strains which are represented with coverages ranging from 3.48× to 2.77×. Characteristic of agricultural soil, the simHC data set contains no dominant strains, and has poor coverage. The highest organism coverage in this data set is at an estimated 0.53×.

#### Evaluation procedure

As previously described, the simulated metagenome data contains three data sets designed to represent three microbial communities of varying degrees of complexity. Here we present the analysis of binning performance on the medium complexity simMC data set, as this serves as a basis for comparison against current compositional binning techniques. Similar to [39], we conduct two tests to evaluate the quality of binning using OFDEG. The first takes only contigs which are greater than 8,000 bp in length - as these were deemed to have a minimal degree of chimerism. The second takes major contigs, which are those that are assembled using at least 10 reads. We use both the assembler output of Arachne [48] and phrap for evaluation, where phrap produces shorter contigs but is deemed more reliable [39]. Contigs generated by Jazz were excluded from the analysis [46]. Similarly, for the purposes of comparison we restrict ourselves to the taxonomic rank of *order*, using NCBI's taxonomy definition. For all tests conducted, OFDEG values were computed on the basis of a 4-mer OF profile for comparison against tetranucleotide frequency (TF) recommended by [46]. The sampling depth was set to 20 and a step size of 10% of the sequence length was used. Each OFDEG value for a sequence is an average over 5 - determined empirically - subsequences which were truncated to the length of the shortest contig for each test, given the criteria of selecting contigs as defined above. All the OFDEG values computed used 80% of the sequence length. In an attempt to increase the discriminatory power of OFDEG for sequence separation, we also consider the effect of OFDEG in conjunction with G-C content as it has been used previously to successfully characterise organisms and maintains the low dimensionality of the proposed feature space.

#### Measuring the accuracy

Accuracy is measured by how well a particular organism is characterised. With respect to each organism, the bin that is identified as containing the maximum number of fragments for that particular organism is considered as its reference bin. Fragments assigned to the reference bin that are of the reference organism are deemed the *True Positives*. Similarly, fragments contained in the reference bin that are not of the reference organism are *False Positives*. Fragments of the reference organism that are located outside the reference bin are considered *False Negatives *and lastly, fragments that are not of the reference organism and located outside the reference bin are considered *True Negatives*. Using these interpretations we use the standard definition of sensitivity and specificity to evaluate the quality of binning.

For the semi-supervised case we look at the label assigned to each fragment and compare this assignment to its true origin. For each class of organism, at a specific taxanomic rank, we look at how many of those fragments have been assigned correctly using the same definitions as for the unsupervised case. Fragments that are not assigned a label are not included in the calculation.

### Unsupervised setting

We first evaluate the fidelity of OFDEG by clustering computed OFDEG values in an unsupervised manner. For compositional methods, the relative abundance of oligonucleotide frequencies and inherent biases therein have been linked to a sequence's phylogenetic origin. Raw OFDEG values are clustered using Partitioning Around Mediods [49], and the average silhouette width is used to compute the most representative number of classes based on the clustering structure.

**Figure 1:**
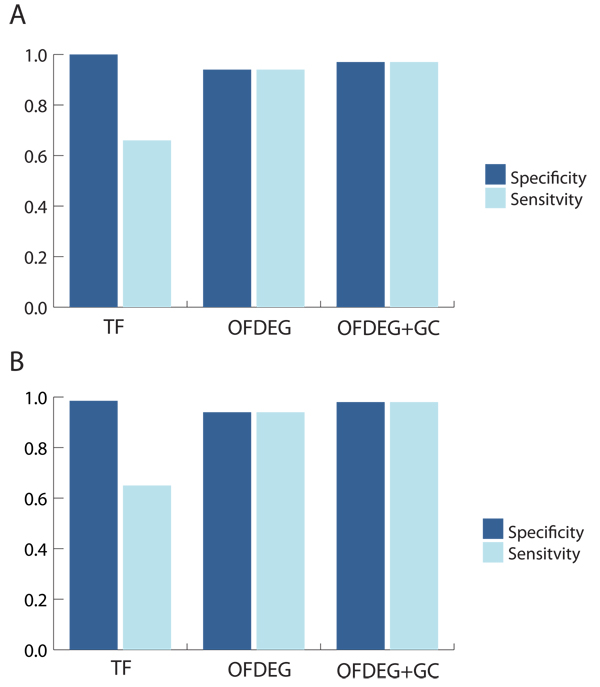
**simMC (≥ 8 kbp): unsupervised setting**. OFDEG, OFDEG+GC and tetranucleotide frequency (TF) comparison using unsupervised methods, for the Phrap (A) and Arachne (B) assemblers. We see that particularly in the sensitivity measure of binning performance, OFDEG features greatly improve on the TF feature space. There is evidence of only a minimal improvement in performance with the addition of G-C content, which demonstrates that OFDEG alone has greater capacity for binning.

**Figure 2:**
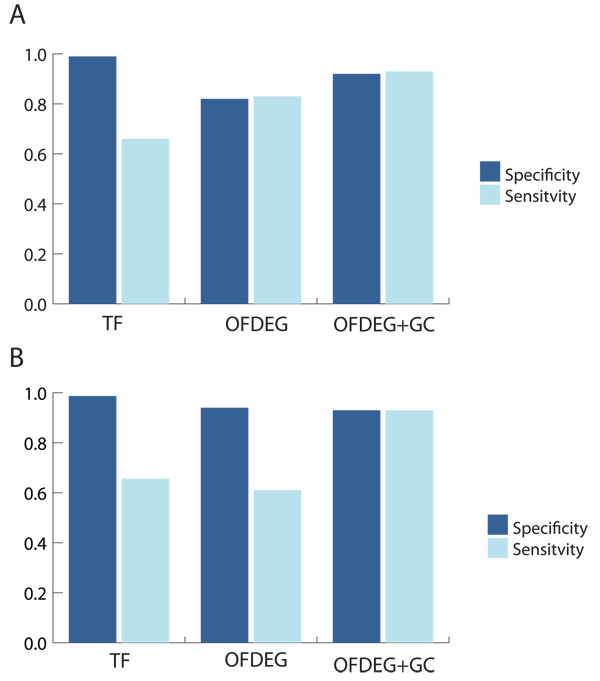
**simMC (≥ 10 reads): unsupervised setting**. OFDEG, OFDEG+GC and tetranucleotide frequency (TF) comparison using unsupervised methods, for the Phrap (A) and Arachne (B) assemblers. Here we see a similar scenario to the 8 kbp tests. In this case, however, augmenting OFDEG with G-C content is much more beneficial, where OFDEG alone appears to offer lower accuracy.

In general, it is clearly evident that clustered OFDEG values in conjunction with G-C content improves on the performance of tetranucleotide frequency alone (Figures 1 &2). The lower sensitivity value for tetranucleotide frequency can be explained by the less defined clusters that result, where fragments that are associated with one genome type are distributed across multiple bins.

As the reference sequence length for OFDEG calculation must be the same for all sequences in order to produce meaningful comparisons, the minimum contig lengths in the 10 reads tests highlighted the benefits of using OFDEG. For the phrap assembled data, the minimum contig length is only 230 bp, yet we see that the binning fidelity is competitive with features that require the entire sequence length. With an increase in minimum contig length for the Arachne assembled data to 1334 bp, again for the 10 reads test, the binning performance increases. This is even more prevalent when considering the 8 kbp tests, which have a minimum contig length of 8 kbp, where a near perfect separation of the two out of three dominant species is observed.

### Semi-supervised setting

In this setting we require the use of a minimal amount of annotated data which we will refer to as labels, a basis for supervised learning. Using labels, we are able to investigate the possibility of a performance increase using existing knowledge of sequenced microbial DNA, an observation described in [46]. Here we also use the S-GSOM algorithm [46]. Fixed-sized SOMs were not used, as the imposition of a fixed sized map may result in incorrect representation of the input space, and subsequently poor clustering. Note, this was used only as a comparison of features, and not a contribution as a novel clustering technique. Similar to [46] we select as labels 10 kbp flanking regions of the conserved 16S rRNA gene, subject to rules also defined therein. OFDEG values are computed for each flanking sequence of 16S rRNA genes found in the genomes used in the simulated data sets. These values were augmented with the processed data sets as *seeds*. Additionally, Topology Type, Similarity Measure, Weight Initialisation Type, Neighbourhood Kernel, Initial Learning Rates, and Training Epochs for S-GSOM were selected to be the same. For comparison, we also evaluate classification performance at cluster percentages (CP) of 55% and 75%, as these parameter settings are recommended in [46].

**Figure 3:**
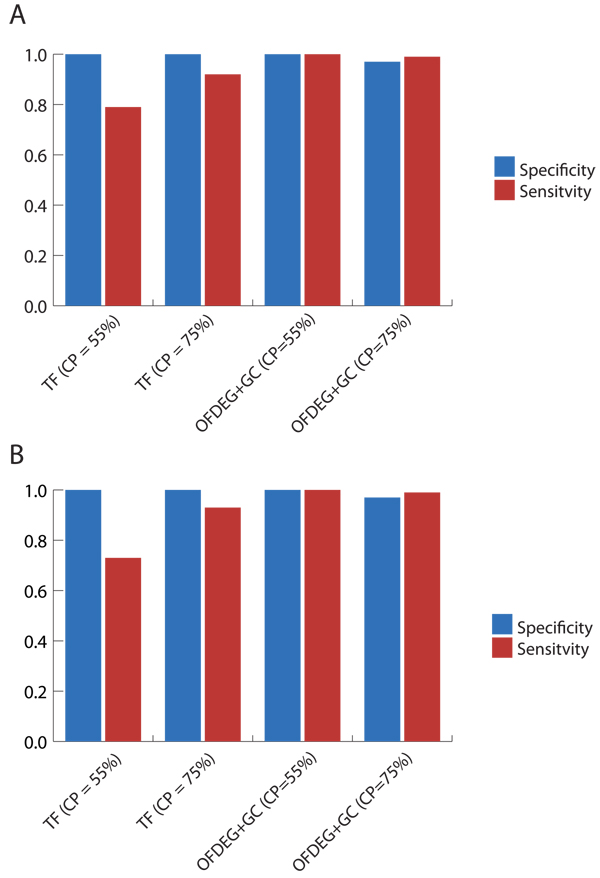
**simMC (≥ 8 kbp): semi-supervised setting**. OFDEG+GC and tetranucleotide frequency (TF) comparison using semi-supervised methods, for the Phrap (A) and Arachne (B) assemblers. As anticipated, the addition of known seeds improves the accuracy of binning using OFDEG in a purely unsupervised manner. However, we note that at higher values of CP the classificaiton performance will degrade due to an increasing number of incorrect assignments made on ambiguous fragments.

**Figure 4:**
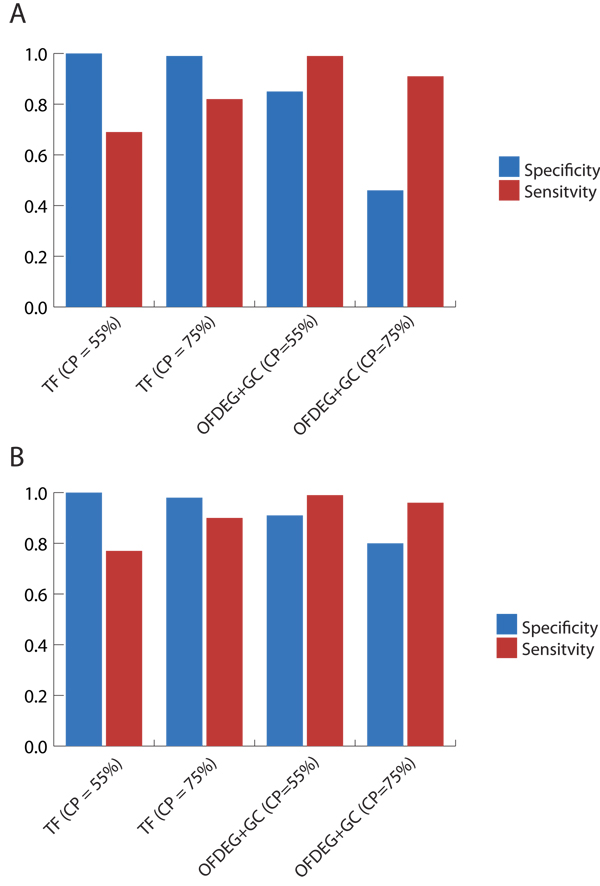
**simMC (≥ 10 reads): semi-supervised setting**. OFDEG+GC and tetranucleotide frequency (TF) comparison using semi-supervised methods, for the Phrap (A) and Arachne (B) assemblers. We observe these results to be of a similar trend to the 8 kbp tests, yet we experience a much more profound reduction in specificity on the Phrap data set. We attribute this to the shorter fragments contained in the Phrap data set, which further contributes to the amibiguity in association between genomic fragment and parent organism.

Particularly for the 8 kbp tests, we see an improvement in performance over a purely unsupervised attempt at characterising fragments, achieving a sensitivity and specificity of 1 for both assemblers (Figure 3). As is appreciated in purely clustered data, there will be ambiguity in assignment at the edges of clusters, i.e. does fragment *x*, which lies directly in between the centres of clusters A and B, belong to cluster A or B? In selecting only those sequences that will give confident predictions, avoiding the previously mentioned situation, the accuracy of binning is increased. In this case, the number of fragments assigned are reduced as a result. Tests using OFDEG for semi-supervised classification at higher CP values (Figure 4) showed a decrease in accuracy, as spurious assignments were made. The increase in performance experienced by S-GSOM using tetranucelotide frequency, on the other hand, can be explained by seed location within a fully trained map.

To be able to use semi-supervised learning, we require an indication of the diversity of the metagenome, perhaps through targeted sequencing of the 16S ribosomal RNA. Given this, the flanking sequences are used as reference classes for binning. However, reference genomes would unlikely be available, given the current knowledge of microbial diversity. The accuracy is then determined by the correct identification of the taxa that is actually present in the data set. This, however, is not a requirement for binning, but merely a means to get a sense of species composition. Binning is still able to proceed without such *a priori *knowledge.

### Overall perfomance and discussion

In order to capture the overall relative performance of both OFDEG and TF feature spaces, we compute the discriminant ability of each using averaged sensitivity and specificity values over all tests conducted. With reference to (Table 1) we see that both the unsupervised and semi-supervised methods which use OFDEG+GC as a feature space perform best overall with respect to the four different simMC tests. Though the semi-supervised method outperforms the unsupervised method, the average number of assignments made by the unsupervised variant is far greater. If labels are available, we are able to classify fragments with approximately 96% accuracy. However, in the case where labels are not present, unsupervised methods can be applied using sequence OFDEG values with 95% accuracy, but at the same time with higher coverage: 97.33% of sequences in the data set as opposed to 63.65% for the semi-supervised case.

**Table 1:** OFDEG values for genomes sourced from NCBI.

Organism	100 bp	200 bp	40,000 bp
Acinetobacter sp. ADP1	-0.09469 ± 0.0057	-0.07171 ± 0.0053	-0.03765 ± 0.0010
Bacillus anthracis Ames 0581	-0.09552 ± 0.0058	-0.07474 ± 0.0067	-0.04156 ± 0.0014
Prochlorococcus marinus MIT 9211	-0.09507 ± 0.0049	-0.07274 ± 0.0042	-0.04361 ± 0.0045
Streptococcus pneumoniae R6	-0.09439 ± 0.0048	-0.07250 ± 0.0045	-0.03773 ± 0.0010
Shigella flexneri 2a str. 2457T	-0.09326 ± 0.0044	-0.07058 ± 0.0030	-0.03486 ± 0.0005
Escherichia coli str. K12 substr. W3110	-0.09310 ± 0.0039	-0.07054 ± 0.0046	-0.03498 ± 0.0006
Thermoplasma volcanium GSS1	-0.09296 ± 0.0047	-0.07194 ± 0.0050	-0.03639 ± 0.0010
Pseudomonas aeruginosa PA7	-0.09655 ± 0.0067	-0.07664 ± 0.0064	-0.04511 ± 0.0045
Xylella fastidiosa M23	-0.09410 ± 0.0049	-0.07097 ± 0.0036	-0.03572 ± 0.0014
Maricaulis maris MCS10	-0.09540 ± 0.0057	-0.07297 ± 0.0043	-0.04096 ± 0.0019

Computed OFDEG values using 5-mer oligos for 10 microbial genomes selected arbitrarily from NCBI. The values listed here represent the sample mean plus or minus the sample standard deviation, averaged over 100 sampling sites over the entire length of the genome. The differences reflect the strength of the signal in relation to sequence length.

An important consideration is data dimensionality. For instance, projecting a sequence into TF space results in the generation of input vectors of dimension 256 for each sequence in the data set. With a large number of sequences, the computational cost of clustering such data will be taxing, even more so with higher order oligos. Alternatively, OFDEG is a consistent, one-dimensional feature space - two at most, when used in conjunction with G-C content - irrespective of the underlying word size, which will be beneficial in the initial analysis of metagenome data. We appreciate that seeding posseses the capacity for a more accurate classification of environmental DNA fragments. However, care should be taken when using this approach. As noted earlier, this setting relies on the premis that the 16S rRNA flanking sequences are representative of the biota in the sample and are available for each species in the metagenome. Using seeds which are incorrect will degrade the classification performance, even if the clustered data is structured correctly.

Practical applications of using OFDEG should take into consideration the following. Current next-generation sequencing technologies are producing output at higher rates and shorter read lengths. The method proposed operates under the assumption that some assembly has been carried out on the raw sequencer output. Akin to compositional methods, sufficient sequence length is required to make inferences based only on the ordering of base-pairs. However, this compositional feature appears to make a stronger association between phylogeny and sequence composition given shorter strecthes of DNA. Of additional importance are repeated regions in genomic DNA. These will be captured and reflected in the computed OFDEG values, which will be lower in comparison to other seqeuneces. For the detection and removal of, say, redundant repeats, a preprocessing tool could be used to remove these prior to OFDEG computation. This, however, is beyond the scope of this work. We emphasise that this work serves to describe the observation of a characteristic linear gradient and its potential application. Although we are unable to fully explain its theoretical underpinnings or provide an in-depth biological interpretation, we are empirically able to show that it does have links to microbial phylogeny.

## Conclusion

Here we have presented a novel representation of short DNA sequences, derived from oligonulceotide frequency profiles, which allows for the phylogenetic characterisation of relatively short sequences on the basis of sequence compostion alone. Although we have found that microbial phylogeny is potentially captured in OFDEG, we aim to develop a theoretical framework as well as ellicit its biological meaning. Unsupervised clustering revealed OFDEG related features performed better than standard tetranucleotide frequency in representing a relevant organism specific signal. The extension to a semi-supervised paradigm again demonstrated an improvement in binning performance when using OFDEG values. In light of the issues faced with semi-supervised classification of OFDEG values, an interesting prospect for future work is the analysis of seed selection and its influence on the accuracy of fragment classification, especially for data sets which contain short contigs.

Expressing each sequence in an appropriate feature space is more beneficial than developing intricate machine learning methods that wrap around feature representations that do not capture phylogenetic signals in short sequences. There is a pressing need to break away from reliance on assemblers that were designed to handle single genomes, built without consideration for processing significantly different, heterogeneous metagenome sequence data. Addressing the fundamental issue of a suitable representation for short DNA sequences has shown potential as a first step toward unbiased, database-independent characterisation of metagenome data and novel microbiota.

## Methods

### Defining OFDEG

In this section we describe what we mean by OFDEG, and subsequently how it can be used as a genomic signature with particular emphasis on its application to the analysis of a metagenome. In order to describe OFDEG we firstly outline the procedure for computing the oligonucleotide frequency (OF) profile of a DNA sequence from which OFDEG is observed. The OF profile of a DNA sequence captures the relative abundance of all possible enumerations of oligos, or words, of a predefined length. For example, capturing words of length *m *would constitute 4^*m *^possible combinations of the bases A, C, G and T. As a DNA sequence is traversed, using a sliding window and a user-specified step size, each word that is encountered adds to the total count of that word in the sequence's OF profile. What results is a histogram depicting the relative abundance of all words present in a sequence. Normalisation of the OF profile by sequence length allows sequences of different lengths to be compared. Comparisons can be made using a Euclidean distance metric, for instance. Now, sequences that have similiar OF distributions are inferred to be phylogenetically similar up to some taxanomic rank. The phylogenetic signal that is captured using length-normalised OF profiles is weakly present in sequences of 1,000 base pairs and greater. It is this common signal between genomes and its sub-sequences that allows the association between a DNA fragment and its source organism.

#### Observation of a linear relationship between error and sub-sequence length

**Figure 5:**
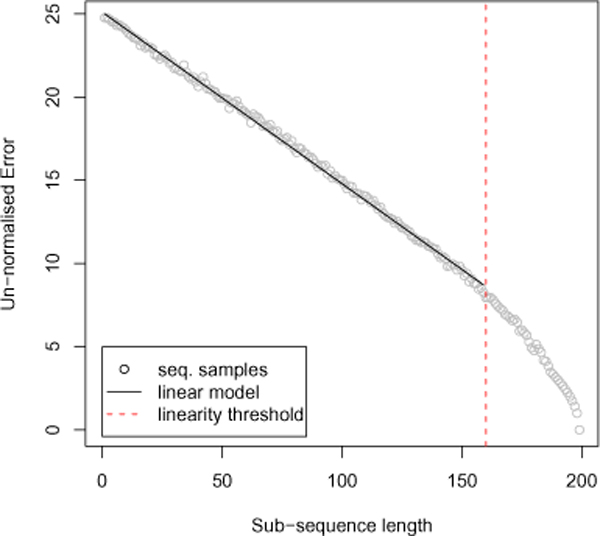
**OFDEG**. The linearity observed in the reduction of error for an artificially generated 200 bp DNA sequence, sampled at fixed intervals along its length. Each sampled error represents the Euclidean distance between the un-normalised OF profile of the entire sequence and that of a shorter sub-sequence. The reduction in error is linear for sub-samples that are less than approximately 80% of the sequence length, was similarly observed for sequences greater than 200 bp (data not shown). The gradient of this linear region is what we refer to as OFDEG.

The error between length-normalised OF profiles of entire genomes and that of short sequences is considerable, so much so that their association is blurred in the presence of other genomic sequences from isolate organisms. If we take a closer look at this representation we encounter a fundamental feature of OF profiles that gives rise to a secondary signal, which potentially breaks through the 1,000 base pair "barrier" [16]. As previously mentioned, the relative abundance of oligos is what differentiates between various species. Length normalisation ensures that the abundance of oligos in long sequences is comparable to that of a short sequence, assuming of course that the effects of sequence polymorphism is minimal. If this step is omitted, and the comparison is made between such sequences using a Euclidean distance metric, the error would be significantly more substantial. Counter-intuitively, this is where our proposed signal is found. It is by this means that we attempt to capture a conserved global signature, if one actually exists. The idea behind OFDEG is a simple one and is obtained as follows. If we take an entire genome, for example, we are able to easily compute its OF profile. If we then take a short sub-sequence from anywhere along the genome, we are able to compute its OF profile also. According to the above, the Euclidean distance (error) between the two would be large. Nevertheless we take note of this error. We then take another sub-sequence but of increased length, again from anywhere along the genome. Trivially, the error between this new sequence's OF profile and the genome's OF profile would be reduced. We continue this process until our sub-sequences are of the same length as the genome itself, while keeping track of the error at each sub-sampling event. If we plot the error as a function of sub-sequence length, we arrive at a linear reduction in error up to a certain sub-sequence length. The rate of error reduction, within the bounds of the linear region, is the sequence's OFDEG value. We believe that the linearity is not a biological phenomena associated with peculiarities in genome composition, but rather a function of OF profiles in general. However, biological rationale for the variation of gradient values is not yet understood. Figure 5 shows the result of sub-sampling an artificially generated DNA sequence (the generation and use of artificial sequences will be described later).

#### Validitiy of the linear model

**Figure 6:**
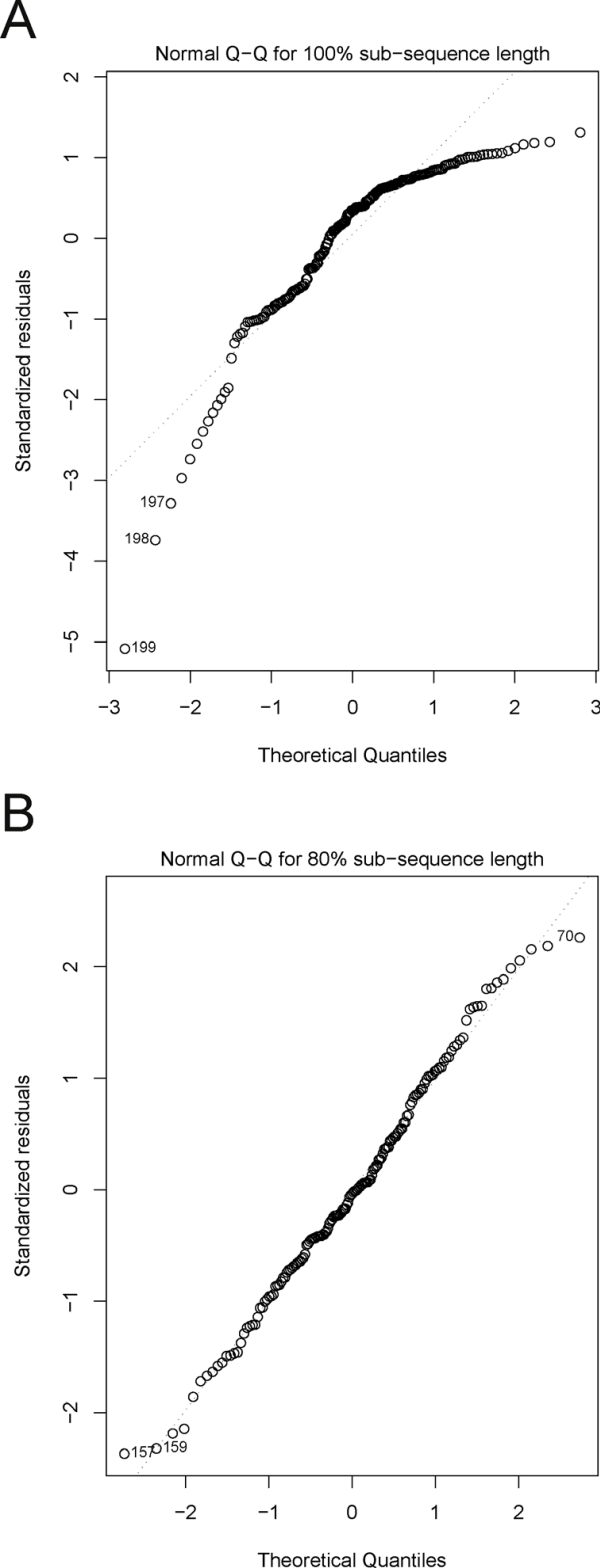
**Linear model validation for 100% and 80% sequence length**. Linear model validation at 100% sequence length (A), and with the linearity threshold applied to 80% of the sequence length (B). We see that omitting the remaining 20% of a sequence results in a better fit of the linear model.

In actuality, it is empirically observed that this linearity is limited to approximately 80% of the sequence length. Normal Q-Q plots for applying a linear model to 100% of the sequence versus only 80% of the sequence length are shown in Figure 6. It is clear that linear regression applied to 80% of the sequence more accurately captures the reduction in error, as opposed to applying the model to the entire sequence.

#### Step size and sampling depth

**Figure 7:**
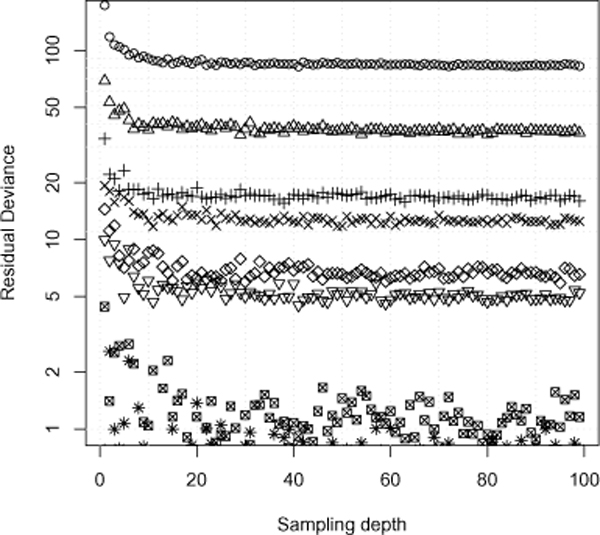
**The effect of sampling depth**. Here we plot the residual deviance of the linear model applied to the error gradient of a 200 bp fragment for various step sizes. Each curve corresponds to a particular step size for a fixed sequence length. Notably, the effect of step size is independent of sampling depth, and further a sampling depth of 20 gives a good model for the reduction in error over sequence length for sequences in the order of magnitude considered in the benchmarks. Intuitively, larger sequence lengths will require greater sampling depths.

Given the above description of how we arrive at an OFDEG value for a sequence, we are now left with parameters which require definition. The two parameters that are of significance here are the *sampling depth *and *step size*. The sampling depth refers to the number of equal length sub-sequences randomly selected from the entire sequence. The average of these values are used to determine the error value for a particular sub-sequence length. The step size is the change in sub-sequence length from one sampling instance to the next. Empirically, we found that a sampling depth of 20 provides a good fit of the error reduction rate, independent of step size (Figure 7). Note that this is not universal across all sequence lengths. As the entire sequence length is increased the number of required samples per sampling instance increases. For the sequences considered here, they are in the order of 250-1800 bp, so a sampling depth of 20 or more provides a good approximation to a sequence's OFDEG value. It is observed that the sampling depth has greater influence on the error gradient than the step size. Nevertheless, a step size less than approximately 10% of the sequence length will offer lower variance in the OFDEG estimate.

### Computation of OFDEG

The method by which an OFDEG value is compuated for a sequence is described as follows. Note, the following procedure assumes a user-selected word-size *m*, step-size *t*, sampling depth *d*, minimum sequence cutoff *c *(which corresponds to the length of the shortest sequence in the data set) and cutoff re-sampling depth *r*_*c*_.

1. Start with a sequence *S*_*L *_of length *L*. If *L *> *c*, truncate *S*_*L *_to *S*_*c *_from a random location anywhere along the sequence.

2. Take a sub-sequence *s*_*i *_of length *i *= *m *(initially) from a random location in *S*_*c*_. Compute the OF profile of *s*_*i *_and of *S*_*c *_and compute the Euclidean distance between them, and let this disparity be referred to as the *error *and denoted *e*_*i*_. For the same sub-sequence length, repeat the procedure *d*-times. Taking the average of all the error values, , gives one sample point.

3. Increase the sub-sequence length by *t*, i.e. *i *+ *t*. Repeating the above procedure gives , and so on, until we have obtained  - note that  = 0 as there is no disparity between *s*_*c *_and *S*_*c*_.

4. Performing linear regression on all 's for *i *∈ [*m*, *αc*] gives an estimation of a sequence's OFDEG value.

5. If *L *> *c*, go back to step 1 and repeat steps 1 to 4, *r*_*c *_times, to obtain a more robust OFDEG estimate.

As previously described, the predefined paramters were empircally determined, most notably the range of *α *being approximately (0, 0.8].

### Capturing a characterestic of a sequence

According to our observation, if the OF profile is sparse, then most of the time we have captured predominantly the same oligo; in such a case, the error gradient will be strongly linear and closer to 1. If on the other hand, the OF profile is wildly distributed, the linearity would be relatively weaker, and the gradient lower. The distribution of an OF profile is in turn thought to be governed by the complexity of the sequence. Sequences that exhibit high degrees of polymorphism will have more dispersed oligo counts for the same sequence length than a simple sequence, and will therefore yeild lower error gradients. We elaborate on this concept by simulating DNA sequences of controlled complexity. We construct these aritificial sequences by taking an arbitrary base, say *A*, and allowing the next base to be one of *G*, *C *or *T *with probability equal to *p*.

**Figure 8:**
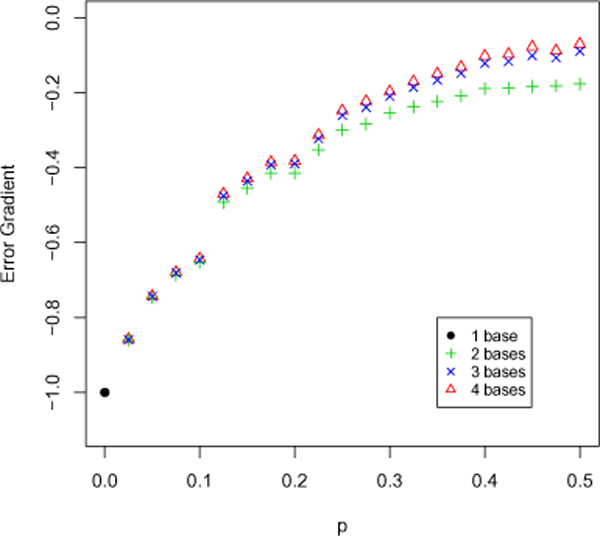
**OFDEG as a function of sequence complexity**. The effect on the reduction in error rate when varying the complexity of a sequence, represented here by the probability of base change *p*. As expected, we see that for sequences with more widely distributed OF profiles, a lower error gradient results as is seen with sequences composed of 4 bases as opposed to say 1 to 3 bases. Further, an increase in the complexity of the sequence, by allowing numerous base changes over the sequence length we also arrive at a lower error gradient.

The generation of sequences with varying number of bases allows further control of the distribution of oligo counts, when the same word size is used. A greater number of bases will distribute the OF profiles more widely, for a given sequence length. For example, a 4-mer OF profile generated using a sequence with only three bases will result in zero counts for oligos containing the missing base. This forcibly controls the width of the OF profile distribution. Based on these observations (Figure 8) there is an apparent correlation between the composition of a DNA sequence and its inherent complexity. We see that for sequences of higher complexity, the rate of error reduction is quite low, which agrees with our initial assumptions.

#### Phylogenetic relevance

To examine the extent to which OFDEG is applicable to microorganism characterisation, we sub-sampled fixed length sequences extracted from 10 microbial genomes (NC_005966.1, NC_007530.2, NC_009976.1, AE007317.1, AE014073.1, AP009048.1, BA000011.4, CP000744.1, CP001011.1 and NC_008347.1); the results of which are shown in (Table 2). The computed OFDEG values correspond to the average error reduction rate over 100 randomly selected sites along the genome, for sequences of length 100, 200 and 40,000 base pairs. The ability to capture the genomic complexity is not universal across all sequence lengths. We ultimately see that 100 bp sequences are difficult to characterise, and there appears to be no strong correlation between OFDEG values and phylogeny. Increasing the sample length to 200 bp increases the resolution at which fragments can be associated to a particular genome; the phylogenetic signal becomes much stronger here. For example, considering the strains *Escherichia coli str. K12 substr. W3110, Shigella Flexneri 2a str. 2457T *and *Xylella fastidiosa M23*, it is clear that their mean OFDEG values are closely matched. If we examine their phylogeny, we see that the E-coli strain and the Shigella strain are both of the family *Enterobacteriaceae*, which are reflected in their relative OFDEG values in relation to the Xylella strain which is of the family *Xanthomonadaceae*. Collectively, though, all three strains fall under the class of *Gammaproteobacteria*, which is again reflected in their mean OFDEG values in relation to other genomic fragments of 200 bp.

**Table 2:** Overall ranking in terms of discriminant ability.

	Type	avg. assigns.	avg. *S*_*p*_	avg. *S*_*n*_	Discriminant ability
OFDEG+GC (CP = 55%)	semi-supervised	63.65%	0.9400	0.9950	0.9675
OFDEG+GC	unsupervised	97.33%	0.9513	0.9525	0.9519
TF (CP = 75%)	semi-supervised	83.44%	0.9925	0.8925	0.9425
OFDEG+GC (CP = 75%)	semi-supervised	77.75%	0.8000	0.9625	0.8813
TF (CP = 55%)	semi-supervised	69.28%	1.0000	0.7450	0.8725
OFDEG	unsupervised	97.34%	0.9100	0.8300	0.8700
TF	unsupervised	97.34%	0.9905	0.6565	0.8235

Discriminant ability is given by the average of the sensitivity (*S*_*n*_) and specificity (*S*_*p*_) values. In this case, we take the average of the average specificity and sensitivity over all tests conducted. We see that both the unsupervised and semi-supervised methods which use OFDEG+GC as a feature space perform best overall with respect to the simMC tests. Though the semi-supervised method outperforms the unsupervised method, the average number of assignments made by the unsupervised variant is far greater.

Notably, there will be unavoidable inter-genomic overlap in the computed OFDEG values, depending on the location of the sequence along the genome. In particular, species with a high degree of polymorphism will be difficult to characterise in the presence of other species. This is observed at a more significant level for smaller oligos (less than a word size of 4). Where we see outliers that are of higher gradient, we have sampled a genomic sequence of lower complexity and conversely outliers of lower gradient are regions in the genome that have higher complexity. We can attribute this to varying degrees of sequence polymorphism, and possibly even due to horizontal gene transfer events. The variability of the genome is captured more concisely in longer DNA fragments, as is expected. Shorter fragments exhibit greater variations, and are more susceptible to noise. However, evidence remains that there is still a phylogenetic signal present for relatively short sequences. The ability to capture overall complexity of short microbial DNA sequences could be associated with the high density of open-reading frames, which leaves little room for highly variable inter-genic regions that are more susceptible to the pressures of environmental stresses and evolutionary changes.

## List of abbreviations used

OFDEG: Oligonucleotide Frequency Derived Error Gradient; OF: Oligonucleotide Frequency; TF: Tetranucleotide Frequency; CP: Cluster Percentage.

## Competing interests

The authors declare that they have no competing interests.

## Authors' contributions

IS conceived of the oligonucleotide-frequency derived error gradient, conducted the benchmarking tests and drafted the manuscript. SKH participated in the analysis of results and provided assistance in drafting the manuscript. Both authors approved the final manuscript.

## Note

Other papers from the meeting have been published as part of *BMC Bioinformatics* Volume 10 Supplement 15, 2009: Eighth International Conference on Bioinformatics (InCoB2009): Bioinformatics, available online at http://www.biomedcentral.com/1471-2105/10?issue=S15.
